# Mitral leaflet dynamics in ischemic mitral regurgitation using high resolution MRI

**DOI:** 10.1186/1532-429X-14-S1-W57

**Published:** 2012-02-01

**Authors:** Melissa M Levack, Walter R Witschey, Jeremy R McGarvey, Norihiro Kondo, Gerald A Zsido, Joseph H Gorman, James J Pilla, Robert C Gorman

**Affiliations:** 1 University of Pennsylvania, Philadelphia, PA, USA

## Background

Leaflet malcoaptation in ischemic mitral regurgitation (IMR) is thought to be caused by a combination of annular dilatation and leaflet tethering due to subvalvular ventricular remodeling. Despite this, little is known regarding leaflet dynamics in IMR. Using high temporal resolution MRI we describe, for the first time, the influence of post infarction ventricular remodeling on leaflet motion.

## Methods

A porcine model of IMR was used. Using direct coronary ligation, five animals were subjected to a posterior myocardial infarction. Eight weeks post infarction animals underwent 4D time-resolved, flow sensitive MRI imaging, 3D MRI cine imaging and 2D phase contrast, high temporal resolution imaging of the mitral valve, left ventricle and aortic outflow tract. Three naïve animals underwent identical imaging protocols to serve as controls. Net mitral and aortic transvalvular flows were determined as well as left ventricular volumes and ejection fraction. Anterior to posterior mid-leaflet tip distances were measured throughout the cardiac cycle for quantification of temporal leaflet dynamics. Statistical significance was computed by ANOVA.

## Results

**Figure 1:**
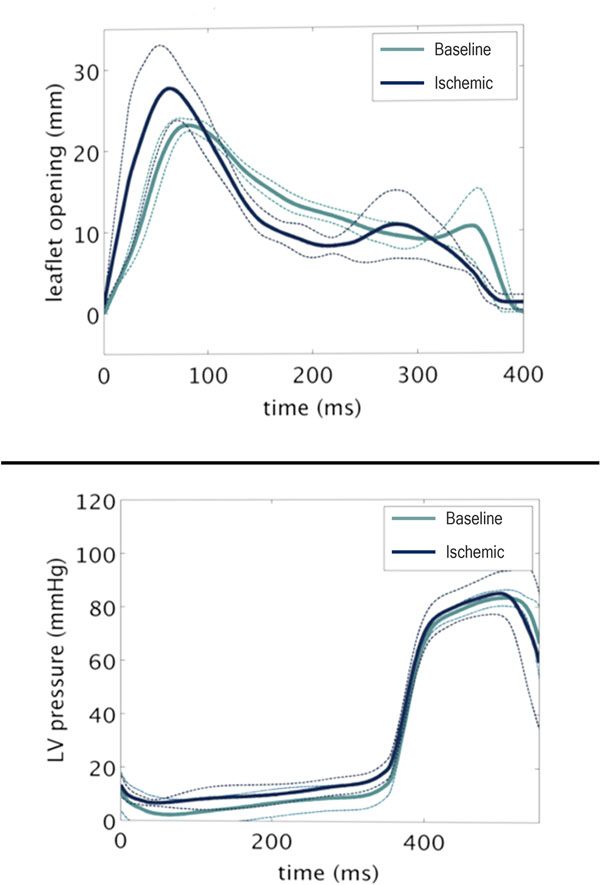
(Top panel) Temporal dynamics of mitral valve leaflet opening with mean values (solid lines) and standard deviation (dotted lines). Animals with ischemic mitral regurgitation demonstrate wider leaflet opening in early diastole. (Bottom panel) Left ventricular pressure throughout cardiac cycle.

Average net flow through the aortic valve was equal to average net flow through the mitral valve in both cohorts. Regurgitant fraction was 11.0 ± 2.3% in the ischemic group. End diastolic volumes for the ischemic group (compared to controls) were 125.6 ± 29.0 ml (55.1 ± 2.5 ml, P=0.01) Similarly, end systolic volumes increased for the ischemic group 87.8 ± 25.9 ml (23.7 ± 3.0 ml, P=0.01). Ejection fraction decreased substantially from 57 ± 5.5% at baseline to 31.2 ± 7.7% (P=0.002) with the development of IMR. Analysis of temporal leaflet dynamics revealed that the early diastolic peak leaflet separation at the A2-P2 scallop was wider in the ischemic group (23 ± 0.1 mm vs 30.1 ± 4.0 mm, P < 0.05). Both cohorts demonstrated interval leaflet closure through mid diastole followed by a blunted enlargement of the opening area during atrial contraction. Figure 1.

## Conclusions

Ventricular dilatation in IMR leads to significant increases in leaflet separation during diastole. The use of high temporal resolution MRI for assessing dynamic leaflet motion can serve as an important addition in future studies looking at the effects of various therapeutic options on valve physiology.

## Funding

This work was supported by grants from the National Heart, Lung and Blood Institute of the National Institutes of Health, Bethesda, MD (HL63954, HL73021 and HL103723). R. Gorman and J. Gorman are supported by individual Established Investigator Awards from the American Heart Association, Dallas, TX.

